# Dataset of bird's eye chilies farm for stereo image semantic segmentation

**DOI:** 10.1016/j.dib.2023.109714

**Published:** 2023-10-23

**Authors:** K.M. Saipullah, W.H.M. Saad, Q.L. Wong, M.S.M. Husni, M.I. Idris, M.S.J.A. Razak

**Affiliations:** a Fakulti Kejuruteraan Elektronik dan Kejuruteraan Komputer (FKEKK), Universiti Teknikal Malaysia Melaka (UTeM), Durian Tunggal, 76100, Melaka, Malaysia; b Strukture Robotics Sdn Bhd, Rekascape, 63000 Cyberjaya, Malaysia; c MSJ Perwira Enterprise, 75460 Duyong, Melaka, Malaysia

**Keywords:** Bird's eye chili farm, Semantic segmentation, Autonomous navigation

## Abstract

This paper presents a dataset of bird's eye chilies in a single farm for semantic segmentation. The dataset is generated using two cameras that are aligned left and right forming a stereo-vision video capture. By analyzing the disparity between corresponding points in the left and right images, algorithms can calculate the relative distance of objects in the scene. This depth information is useful in various applications, including 3D reconstruction, object tracking, and autonomous navigation. The dataset consists of 1150 left and right compressed images extracted from ten sets of stereo videos taken at ten different locations within the chili farm from the same ages of the bird's eye chilies. Since the dataset is used for semantic segmentation, the ground truth images of manually semantic segmented images are also provided in the dataset. The dataset can be used for 2D and 3D semantic segmentation of the bird's eye view chili farm. Some of the object classes in this dataset are the sky, living things, plantation, flat, construction, nature, and misc.

Specifications Table

**Table:** 

Subject	Agricultural Science
Specific subject area	Autonomous Farming
Data format	JPG and PNG
Type of data	Images and mask images
Data collection	The dataset is generated using two High-Definition web cameras that are aligned left and right forming a stereo-vision video capture. The dataset consists of 1150 compressed images extracted from three sets of left and right stereo videos taken at ten different locations within the chili farm from the same ages of the bird's eye chilies. Each video consists of 50 1920 by 1080 pixels images of the video frame and 50 images of the mask according to the classes of the objects. A total of 1000 frame images in JPG format and 150 mask images in PNG format are included in this dataset.
Data source location	Solok Fertigasi by MSJ Perwira Enterprise, Solok Bukit Baru Duyong 75460, Melaka, Malaysia (Lat: 2.208617, Long: 102.288358)
Data accessibility	Repository name: Dataset of Bird's Eye Chilies Farm for Stereo Image Semantic Segmentation [4] Data identification number: 10.17632/3j2vfsfkz5.1 Direct URL to data: https://data.mendeley.com/datasets/3j2vfsfkz5/1 Instructions for accessing these data: Directly download the dataset folder from the given URL.

## 1 Value of the Data

• This data is valuable because it is specific agricultural data, namely the Bird's Eye Chilies farm data that is taken for 2D and 3D image analysis such as segmentation, object recognition, and classification.
• This data can be used in the automation of agricultural activities that require navigation towards the target plants and the understanding of the position and parts of the plants such as harvesting, irrigating, and weeding.
• These data can be reused to examine the performance of the developed algorithms in object recognition, semantic segmentation, and target detection and navigation.

## 2 Data Description

**Fig. 1:**
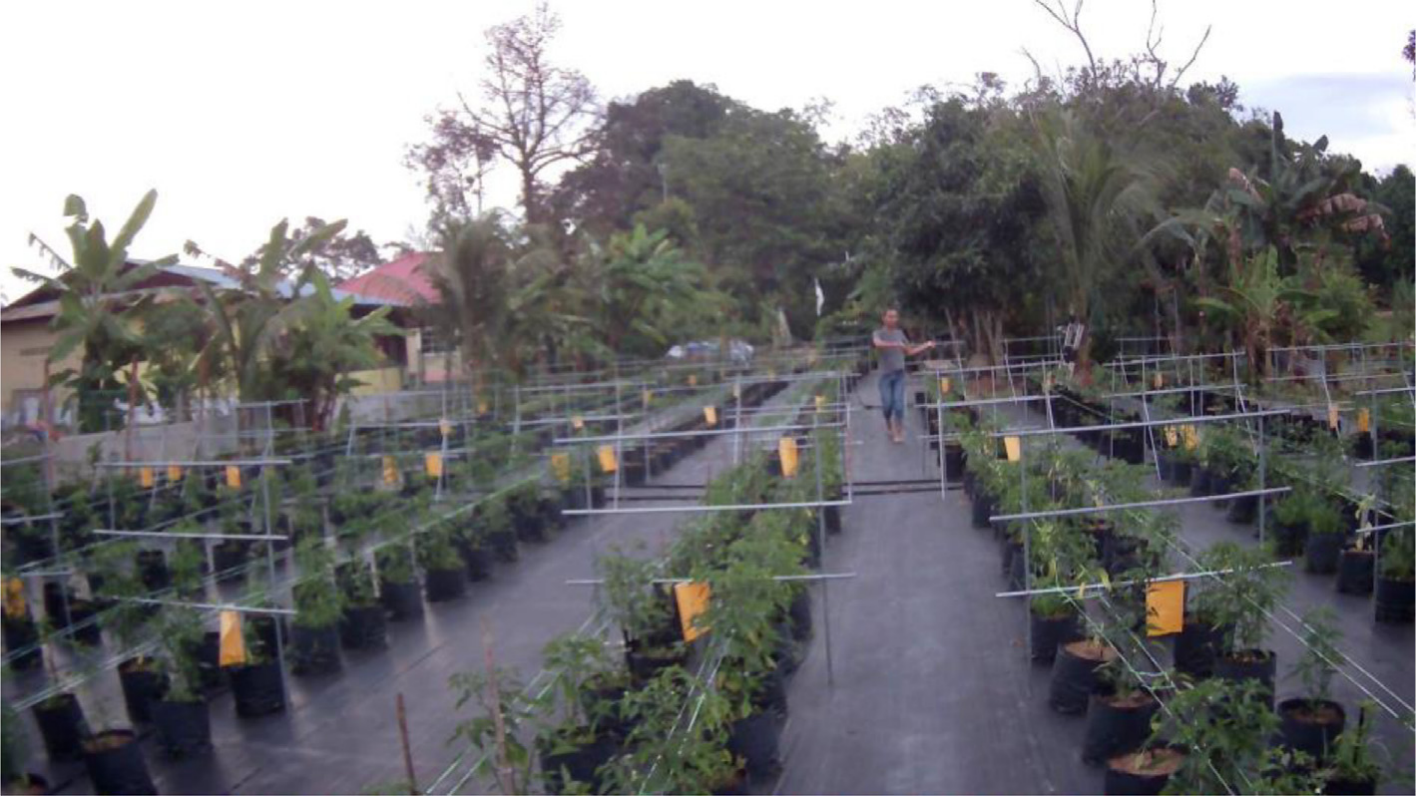
The sample of the video frame in the dataset.

The data consists of frames of ten left and right stereo videos of a five-week-old bird's eye chili tree that were taken on 15^th^ September 2021 in ten different places within the bird's eye chili farm as implemented in [1]. Each video consists of 50 1920 by 1080 pixels images of the video frame and 50 images of the mask according to the classes of the objects. The ground truth image or the mask file needs to be opened as a grayscale image and the pixel value of each of the pixels in the mask image is saved as the object ID as shown in Table 1. The sample of the video frame is shown in Fig. 1. The classes included in the mask are shown in Table 1 according to their object ID which is the pixel value in the mask images. The sample of the mask image of the image in Fig. 1 according to the reference color in Table 1 is shown in Table 2.

**Table 1:** The Object ID and the description of the objects in the dataset.

**Table 2:** Camera parameters information details of the intrinsic and extrinsic resolution.

	Resolution Parameter	Parameter Value	
		Left Camera	Right Camera
	Focal length (pixels): [*f_x_, f_y_*]	[1.5777e+03,1.5763+03]	[1.5744e+03,1.5751e+03]
Intrinsic	Principle point (pixels): [*S_x_, S_y_*]	[1.02026e+03,4.891e+02]	[9.6411e+02,4.81e+02]
	Tangential Distortion: [*X_distorted_, Y_distorted_*]	[-2.8857e-04,6.0283e-04]	[0.0024, -4.2376e-04]

Position and Orientation of Right Camera Relative to Left Camera			

Extrinsic	Rotation of Right Camera (radians): *R*	[1,-0.0114,-0.0055;0.0114, 1,-0.0045;0.0056,0.0044,1]	
	Translation of Right Camera (millimeters): *t*	[-1.1280e+02,-1.61735,2.2989]	

**Fig. 2:**
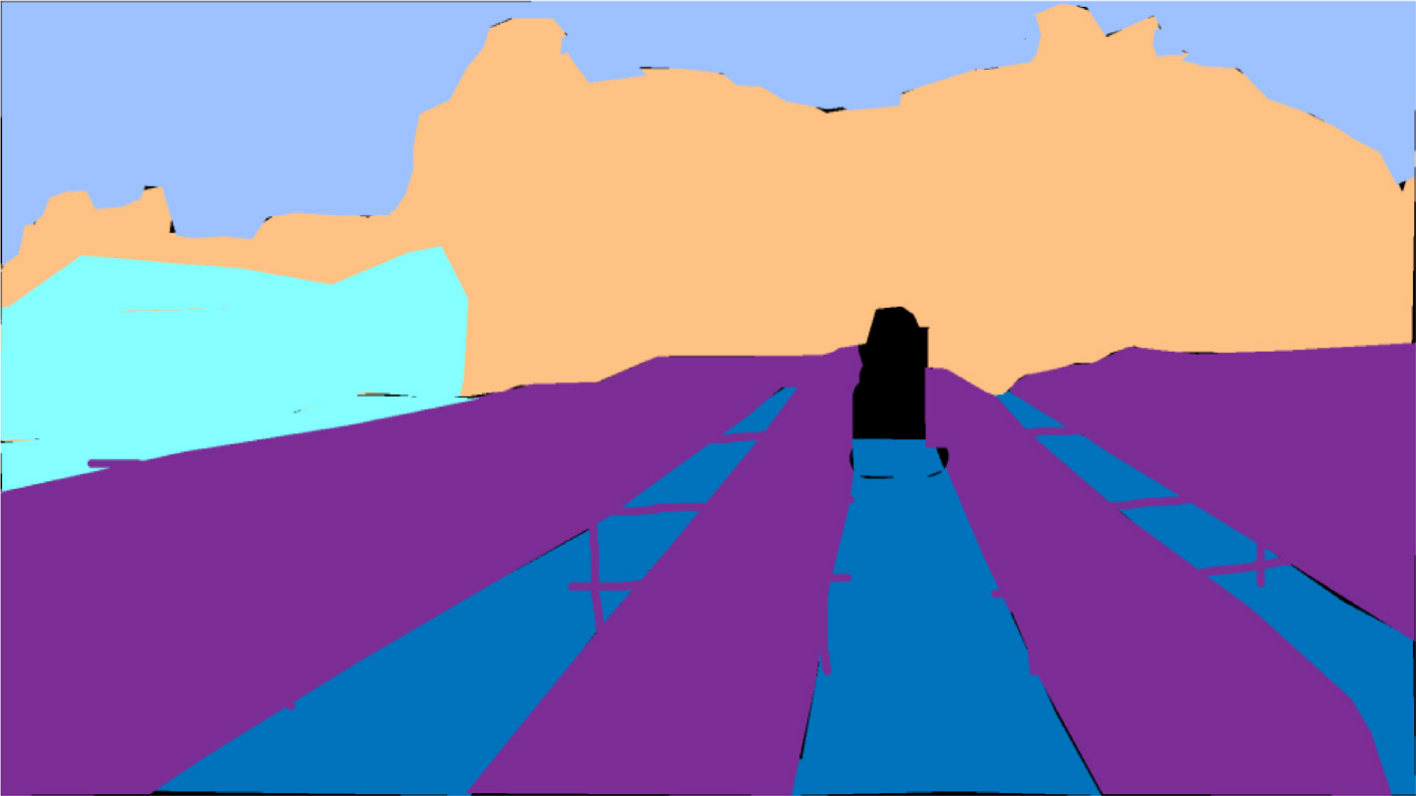
The sample of the semantic segmentation ground truth or mask image of a video frame in the dataset.

**Fig. 3:**
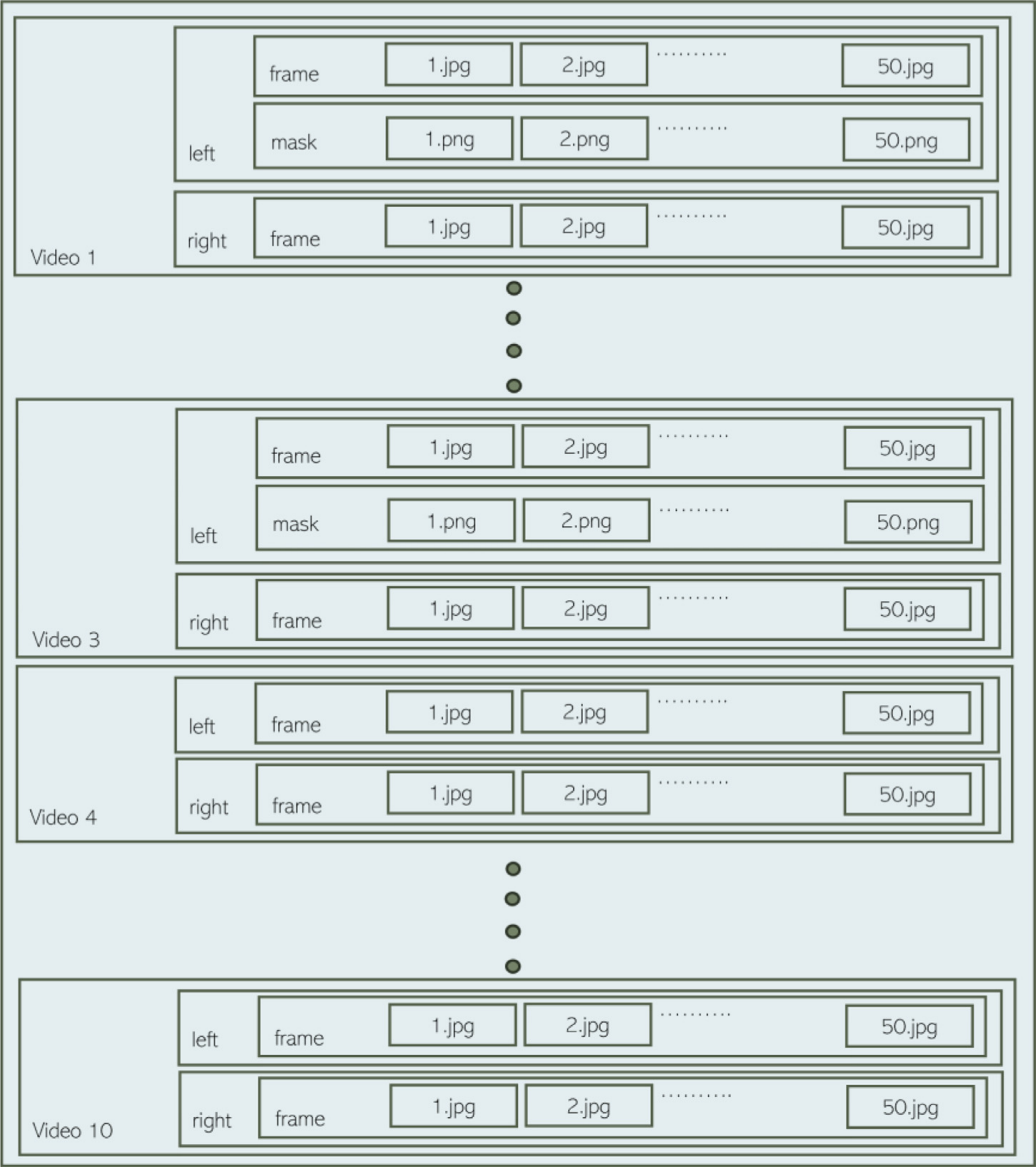
The dataset's folder structure.

The frame images are in JPG format while the mask is in PNG format. Altogether there are 1150 images of video frames and masks included in this dataset. As shown in Fig. 3, there are ten folders in the dataset that are named from *“Video 1”* to *“Video 10”*. Each of the folders consists of 2 folders called *“left”* and *“right”*. In the left folder for *“Video 1”, “Video 2”* and *“Video 10”*, there are two folders named *“frame*” and *“mask”*, respectively. Each file in the frame and mask folder is named as continuous numbers from 1 to 50 followed by the file format. The mask image for a particular frame image will have the same filename as the frame image's filename. For example, the mask for the frame image of *“1.jpg”* is saved in the mask folder and named *“1.png”*. However, in the *“right”* folder for *“Video 1”* to *“Video 10”* and the *“left”* folder for *“Video 4”* to *“Video 10”* only consists of a *“frame*” folder without the *“mask”* folder. This is because, currently, the ground truth images for semantics segmentation for the aforementioned videos as shown in Fig. 2 are not yet available.

## 3 Experimental Design, Materials and Methods

**Fig. 4:**
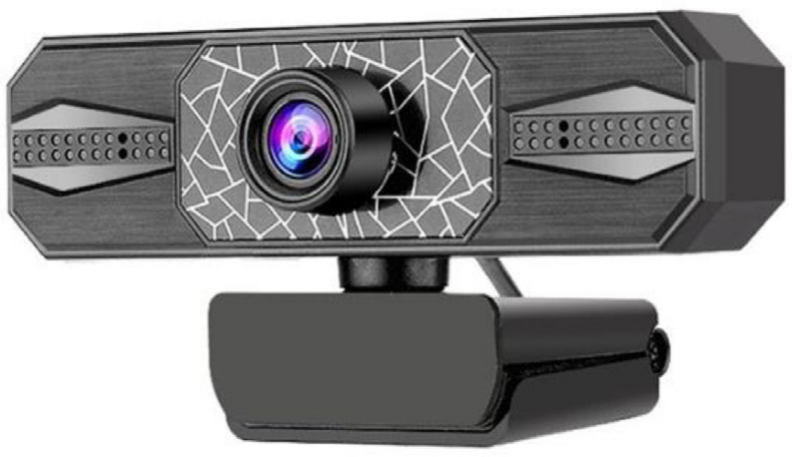
The web camera.

**Fig. 5:**
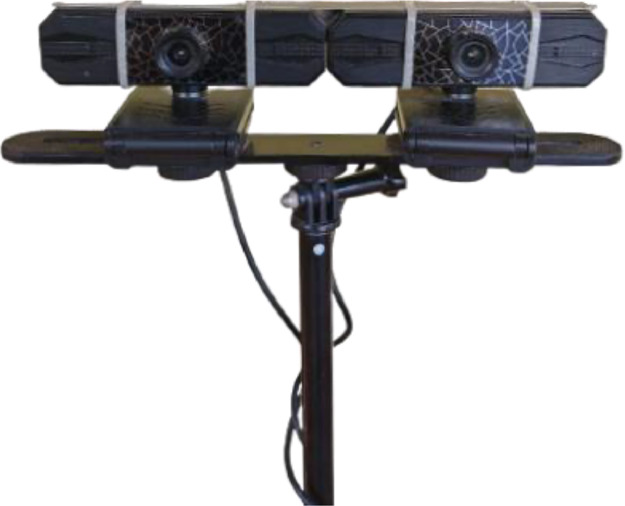
The stereo camera setup.

For the image capture, a high-definition web camera is used as shown in Fig. 4. The camera uses CMOS optical sensor, and the maximum resolution is 1280 by 960 pixels. The default frame rate for the camera is 30 frames per second. To capture the video in stereo, two same cameras are stacked side by side on pole as shown in Fig. 5.

**Fig. 6:**
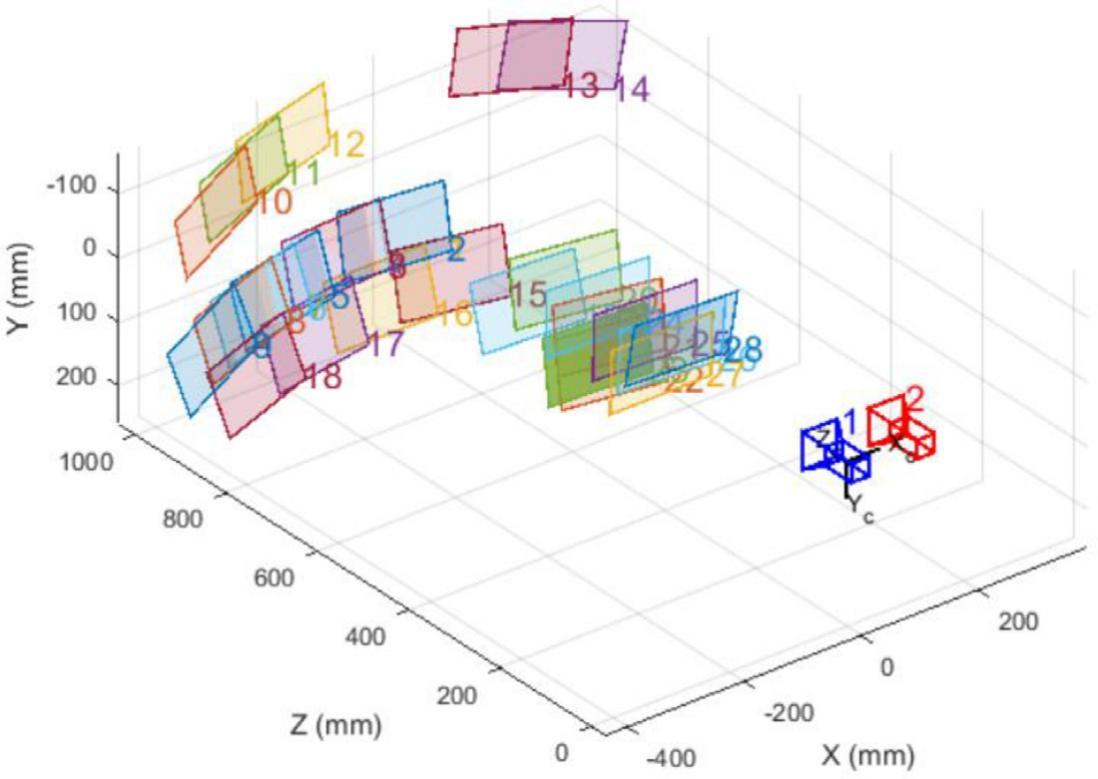
Illustration of intrinsic resolution of the checkerboard pair image.

Camera calibration for stereo vision is done in MatLab using a standard toolbox established in [2,3]. By using 30 pairs of images generated previously, this image is processed. The checkerboard used is an 8 × 5 rectangular square, and each rectangular square has a physical size of 35 mm × 35 mm in dimension. Table 2 shows the value of the camera parameter with the value of intrinsic and extrinsic resolution and the visualization of the values is shown in Fig. 6.

## Limitations

Although the provided data is captured using a stereo camera setup, in this first documentation, only the left video is included in the dataset. This is because ground truth images are still lacking for stereo image segmentation. Since only the ground truth images for a single camera are available, we only provide the single camera data which is the left camera data only in this dataset. The dataset later will be extended according to our research and the generation of the ground truth image.

## Ethics Statement

The authors have read and followed the ethical requirements for publication in Data in Brief and confirm that the current work does not involve human subjects, animal experiments, or any data collected from social media platforms.

## CRediT authorship contribution statement

**K.M. Saipullah:** Data curation, Writing – review & editing, Software. **W.H.M. Saad:** Data curation, Supervision, Conceptualization. **Q.L. Wong:** Data curation, Writing – original draft, Software. **M.S.M. Husni:** Writing – review & editing. **M.I. Idris:** Writing – review & editing. **M.S.J.A. Razak:** Resources.

## Data Availability

Dataset of Bird's Eye Chilies Farm for Stereo Image Semantic Segmentation (Original data) (Mendeley Data).

## References

[1] K.M. Saipullah , W.H.M. Saad, Q.L. Wong, Z.M. Noh, and M.I. Idris. “Validation and annotation scheme for stereo video dataset of bird's eye chili fertigation farm”.

[2] Bouguet J.-Y. (2022).

[3] bin M. Saad W.H., binti Azhar N., bin Roslan A.F., bin A. Karim S.A., bin A. Mana N., binti M. Saad N. (2018). Automated checkerboard orientation detection for camera calibration. Int. J. Imaging Robot.^TM^.

[4] Saipullah Khairul Muzzammil (2023). Dataset of bird's eye chilies farm for stereo image semantic segmentation. Mendeley Data.

